# AML with Myelodysplasia-Related Changes: Development, Challenges, and Treatment Advances

**DOI:** 10.3390/genes11080845

**Published:** 2020-07-24

**Authors:** Kristin L. Koenig, Kieran D. Sahasrabudhe, Audrey M. Sigmund, Bhavana Bhatnagar

**Affiliations:** 1 Division of Hematology, Department of Medicine, The Ohio State University and The Ohio State University Comprehensive Cancer Center, Columbus, OH 43210, USA; Kristin.Koenig@osumc.edu (K.L.K.); Kieran.Sahasrabudhe@osumc.edu (K.D.S.); Audrey.Sigmund@osumc.edu (A.M.S.); 2 OSU Wexner Medical Center, 320 W 10th Avenue, B307 Starling-Loving Hall, Columbus, OH 43210, USA

**Keywords:** acute myeloid leukemia with myelodysplasia-related changes, secondary AML, CPX-351

## Abstract

Acute myeloid leukemia (AML) with myelodysplasia-related changes (AML-MRC) is a distinct biologic subtype of AML that represents 25–34% of all AML diagnoses and associates with especially inferior outcomes compared to non-MRC AML. Typically, patients with AML-MRC experience low remission rates following intensive chemotherapy and a median overall survival of merely 9–12 months. In light of these discouraging outcomes, it has become evident that more effective therapies are needed for patients with AML-MRC. Liposomal daunorubicin–cytarabine (CPX-351) was approved in 2017 for adults with newly diagnosed AML-MRC and those with therapy-related AML (t-AML), and remains the only therapy specifically approved for this patient population. Other studies have also demonstrated the efficacy of the hypomethylating agent (HMA) azacitidine as upfront therapy for AML-MRC patients, which, to date, is the most common treatment employed for patients unable to tolerate the more intensive CPX-351. HMAs and venetoclax combinations have also been evaluated, but additional studies utilizing these agents in this specific subgroup are needed before conclusions regarding their role in the therapeutic armamentarium of AML-MRC patients can be reached. Currently, many studies are ongoing in attempts to further improve outcomes in this historically ill-fated patient group.

## 1. Introduction

Acute myeloid leukemia (AML) is a disease of the myeloid lineage of blood cells that results in a block in differentiation of myeloid cells and uninhibited growth of leukemic blasts that constrain the growth of normal blood cells [1] Clinical sequelae often include malaise and fatigue, infections, bleeding and/or bruising, and possibly bone pain [1]. AML is the most common acute leukemia in adult patients, with nearly 20,000 estimated new cases in the year 2020 and a median age at diagnosis of 68 years [2]. The disease is classified into multiple biologic subtypes according to genetic abnormalities, including both cytogenetic and molecular changes, degree of differentiation, myeloid lineage involved, and dysplastic changes [3]. Notably, these classifications associate with disease prognosis and predict outcomes for patients [3]. 

AML with myelodysplasia-related changes (AML-MRC) is one such subtype of AML that is estimated to represent a sizeable 25–34% of all AML cases and is more commonly seen in older AML patients, with a median age of 73 years [4,5]. AML-MRC portends a worse prognosis than non-MRC AML with both decreased complete remission rate and overall survival [6]. Furthermore, it is often less responsive to standard intensive induction chemotherapy regimens, likely due to both disease biology and clinical characteristics of the patient population that it affects [6]. The aim of this review article is to discuss the leukemic transformation of MDS and disease behavior of AML-MRC, to discuss the challenges associated with treating patients with AML-MRC, to summarize data from clinical trials that have informed therapeutic approaches for this AML subtype, and to discuss potential future directions.

## 2. Definition and Diagnostic Features of AML-MRC

AML-MRC was first introduced in the 2008 World Health Organization (WHO) classification of myeloid neoplasms and acute leukemia and expanded on the prior category of AML with multilineage dysplasia [5]. The 2008 WHO defined AML-MRC as ≥20% myeloid blasts in the bone marrow or peripheral blood and one or more of the following features: a prior history of myelodysplastic syndrome (MDS) or an MDS/myeloproliferative neoplasm (MPN) overlap syndrome, dysplasia in 50% or more of the cells in two or more myeloid lineages, or myelodysplasia-related cytogenetic abnormalities (Table 1) [5]. In the most recent 2016 WHO classification, the definition of AML-MRC was further revised to exclude patients who had *NPM1* or biallelic *CEBPA* mutations, even in the presence of bone marrow findings demonstrating multilineage dysplasia [3]. This change was based on emerging data showing that multilineage dysplasia in the absence of myelodysplasia-related cytogenetic changes did not appear to associate with poor prognosis in the presence of an *NPM1* mutation or biallelic *CEBPA* mutation [7,8,9]. As such, the presence of del(9q) was also removed as a defining myelodysplasia-related cytogenetic abnormality because its association with *NPM1* and biallelic *CEBPA* mutations [10,11].

With the introduction of next generation sequencing (NGS), our understanding of AML-MRC is again evolving with the discovery of the emerging role that common molecular mutations play in AML-MRC and their impact on prognosis. An analysis by Baer and colleagues sought to classify AML-MRC on the basis of molecular aberrations and were able to detect 96–99% of patients with AML-MRC per WHO definition criteria [12]. Mutations that were highly predictive of AML-MRC were *RUNX1*, *TP53*, *SETBP1*, epigenetic regulators, and splicing factors. Furthermore, the molecular MRC-like pattern was identified in over 10% of patients not classified as MRC per WHO criteria but who experienced a similarly poor overall survival (OS), suggesting the definition of AML-MRC may need expanded to include this molecularly-determined subset as well.

## 3. Leukemic Transformation of MDS to AML

The precise mechanism through which MDS progresses to AML remains unclear. However, several factors have been implicated in this phenomenon [13]. Epigenetic changes including abnormal methylation patterns are seen in all MDS bone marrow samples, with a higher amount of methylated CpG sites in high-risk MDS and MDS transforming to AML; it is thought that methylation at CpG sites gives survival advantage to transformed cells [14]. The bone marrow microenvironment has also been proposed to foster MDS progression to AML through increased angiogenesis, development of marrow fibrosis, and promotion of a pro-inflammatory environment [13]. Imbalance between apoptosis and proliferation can heed AML development, as it has been shown that, at the time of MDS progression to AML, there are increased antiapoptotic and pro-proliferative signals as well as increased expression of bcl-2 (b-cell lymphoma 2; an antiapoptotic protein) [15]. The most predominant molecular theory is the so-called “two-hit” model of MDS progression to AML in which sequential genetic alterations in genes altering cellular differentiation (e.g., *TET2* or *RUNX1*) followed by a second “hit” in a gene impacting cellular proliferation and survival (e.g., *FLT3*, *NPM1*, *IDH1*) eventually result in leukemic transformation from antecedent MDS [16,17].

Recently, newer platforms such as targeted deep sequencing and single-cell sequencing have led to an updated non-linear theory of MDS to AML progression. A compelling study by Chen and colleagues proposes that MDS does not directly evolve into AML, but rather parallel clonal evolutionary changes occur in which pre-MDS stem cells and/or MDS stem cells concurrently develop into two separate cell populations: an MDS population and a separate population of pre-AML/AML-stem cells [18]. The latter, through acquisition of additional molecular mutations, is what eventually progresses to AML. Thus, MDS and AML both arise from the stem cell level. MDS stem cells acquire early initiating mutations (such as in *TET2*, *U2AF1*, and *TP53*) then accumulate additional mutations (such as in *NOTCH2* and *KMT2C*), resulting in subclones that can develop into both MDS blasts and pre-AML/AML stem cells. However, pre-MDS/MDS stem cells require other additional mutations in order to develop into AML blasts (such as mutations in *RUNX1*, *NRAS*, and *NTRK3)*. Chen et al. also show that stem cells harbor a higher complexity of subclonal mutations than blasts cells, and the size of different clones varies from MDS blasts to AML blasts. Therefore, dominant and passenger mutations cannot be determined only on the basis of the size of the clone.

In current practice, targeted NGS studies of the blood or bone marrow blasts is performed in order to determine disease mutations in AML but lacks specificity for being able to differentiate between AML and MDS blast populations. Further work is needed to determine how new models of MDS leukemic transformation will be translated into the clinic.

## 4. Challenges in Treatment for AML-MRC Patients

As compared to non-MRC AML, AML-MRC has, overall, been associated with inferior outcomes such as lower remission rates, ranging between 30–50%, and shorter overall survival [6,19,20,21]. For AML-MRC patients able to receive intensive chemotherapy, the overall response rate (ORR) is 69%, with a complete response (CR) rate of 40–51% [21,22,23]. This is compared to less intensive regimens where the ORR and CR rate are, respectively, 69% and 19–32% with single agent hypomethylating agent, 81% and 38–67% with hypomethylating agent in combination, and 69% and 12–36% with low dose cytarabine therapies [22,24,25,26]. Furthermore, median overall survival for AML-MRC patients is typically only 9 to 12 months even in those patients that are able to tolerate intensive induction chemotherapy, which has traditionally yielded the best outcomes for these patients [19,22,27].

### 4.1. Advanced Age

One of the main challenges of treating patients with AML-MRC is that, compared to non-MRC AML which typically has a flatter incidence of occurrence, AML-MRC tends to occur in an older patient population [19,22,28]. The median age at diagnosis of AML-MRC in general is 70 years old (vs. 67 years old for non-MRC AML) and the median age of diagnosis of AML-MRC with a previous diagnosis of MDS is 74 years old [22]. Patients who are older tend to have more co-morbidities and poorer overall performance status and, thus, are less likely to successfully undergo intensive chemotherapy [1,29,30]. Poorer performance status itself has been shown to be an unfavorable prognostic factor in patients with AML, associated with increase in both short and long term mortality [28]. Patients with AML-MRC, given their older age, higher number of comorbidities, and lower performance statuses, tend to be less likely treated with curative intent intensive chemotherapy regimens as compared to younger patients [31]. 

### 4.2. Prior Treatment History

Another challenge in tailoring treatment for patients with AML-MRC is the high likelihood that many of these patients will have had prior treatment with cytotoxic agents preceding their leukemia diagnosis. Although such patients are technically considered to have therapy-related AML (t-AML), there is significant overlap between t-AML and AML-MRC [32]. A recent retrospective study of 415 patients with AML-MRC summarized the most common therapies given prior to their diagnosis of AML-MRC [22]. In this analysis, 22% of patients had received treatment with hypomethylating agents (HMAs), 3% with ruxolitinib, 1% with lenalidomide, and 1% with 7 + 3 for MDS or MDS/MPN treatment prior to their AML-MRC diagnosis [22]. Prior exposure to these agents has potential to not only impact a patient’s ability to tolerate future intensive chemotherapy regimens but may also result in diminished response to future therapies. For instance, Yang and colleagues were able to show that prior treatment with HMAs can result in up-regulation of immune PD-1 (programmed cell death 1), PD-L1 (programmed cell death ligand 1),-PD-L2, and CTLA-4 (cytotoxic T-lymphocyte-associated protein 4), which may result in resistance to hypomethylating agents in the future [33]. Overall, prior cytotoxic exposure has been associated with lower complete remission rates in AML patients with MRC compared to those with non-MRC AML (54–61% vs. 75%) as well as decreased 1-year overall survival (31–56% vs. 65%) [28].

## 5. Pediatric AML-MRC

AML-MRC is reported to occur in approximately 21% of pediatric AML patients (per 2008 WHO criteria), and tends to decline in frequency with increasing age. In fact, 37.5% of AML patients ≤2 years old were found to have MDS-related cytogenetic abnormalities compared to 20.9% and 18.5% of AML patients 3–14 years old and 15–21 years old, respectively [34,35]. In a cohort of 443 pediatric AML patients in Japan, 39 (8.8%) patients had multilineage dysplasia and 65 (14.7%) patients had myelodysplasia-related cytogenetic abnormalities leading to their diagnosis of AML-MRC [34]. Similarly, in a cohort of 140 pediatric AML patients in Greece, 27 (21.8%) patients had AML with myelodysplasia-related cytogenetic abnormalities, qualifying them for a diagnosis of AML-MRC. In these cohorts, the most commonly occurring cytogenetic abnormalities were complex karyotype (14.5–72.3% of patients), −7/del(7q) (9.2–12.1%), and −5/del(5q) (1.6–9.2%) [34,35]. Outcomes in pediatric patients with AML-MRC tended to be worse than in pediatric patients without AML-MRC, with inferior 3-year overall survival (56.8% vs. 68.9%, *p* = 0.05), 3-year event free survival (EFS; 37.1% vs. 53.8%, *p* = 0.02), and relapse-free survival once achieving a CR (46.9% vs. 62.8%, *p* = 0.06) [34].Furthermore, while CR rate after first induction course is similar between these two patient groups (67.7% vs. 77.4%, *p* = 0.12), patients with AML-MRC demonstrated a worse CR rate after a second induction course (67.7% vs. 85.6%, *p* < 0.01) [34].Interestingly, neither multilineage dysplasia nor myelodysplasia-related cytogenetics were associated with OS or EFS on univariate or multivariate analysis in the cohort of Japanese pediatric AML patients [34]

## 6. Treatment Options for AML-MRC Patients

The treatment of AML, until recently, had remained largely unchanged for decades, and consisted primarily of intensive chemotherapy with a cytarabine and anthracycline-based backbone (“7 + 3”) followed by allogenic stem cell transplant (alloHSCT) for curative intent. Although the overall CR rate for the 7 + 3 regimen is reported to be 60–80%, patients with AML-MRC are reported to have lower CR rates with this regimen, typically between 30–50% [21,23,32]. Given these poor outcomes with standard induction chemotherapy, more effective therapies for AML-MRC have been developed and several more are under investigation.

### 6.1. Liposomal Daunorubicin–Cytarabine (CPX-351)

CPX-351 is a liposomal encapsulation of cytarabine and daunorubicin in a fixed 5:1 molar ratio, being approved by the Food and Drug Administration (FDA) in August 2017 for adults with newly diagnosed AML-MRC and t-AML. To date, it is the only therapy specifically approved for this AML subgroup [21,36]. The improved efficacy of this regimen is believed to be related to its unique formulation as the actual liposome-encapsulated drug delivery method allows for a greater amount of CPX-351 to be distributed into leukemia cells than into normal bone marrow cells, which is not seen with traditional cytarabine or daunorubicin administered as standard intensive 7 + 3 induction chemotherapy [35,36]. The phase 3, randomized, open label trial that lead to the approval of CPX-351 investigated 309 patients between 60–75 years of age with de novo AML-MRC, secondary AML, or t-AML and compared outcomes between patients receiving CPX-351 to those receiving standard 7 + 3 therapy [21]. The CPX-351-treated patients demonstrated improved remission rates (47.7% vs. 33.3% (*p* = 0.016)) and median overall survival (OS) (9.56 months vs. 5.95 months (hazard ratio (HR) 0.69)) compared to those who received 7 + 3. Importantly, survival post alloHSCT was also significantly improved in CPX-351 patients (HR 0.46; 95% CI, 0.24–0.89), indicating that the depth of remission achieved in these patients was perhaps superior to the quality of CRs achieved in 7 + 3 patients.

An exploratory subgroup analysis of this trial in the 246 enrolled AML-MRC patients showed that a higher number of patients with AML-MRC obtained a CR/CRi (CR with incomplete hematologic recovery) with CPX-351 than with standard 7 + 3 therapy (48% vs. 33%, odds ratio of 1.83) [37]. This ultimately translated into a longer median OS of 19.2 months compared to 11.6 months (HR 0.58 (0.34–0.96)) and higher rates of allogeneic transplantation in the CPX-351 group (54% vs. 43%). Similar to the entire study population, AML-MRC patients who received CPX-351 also experienced a longer median OS following alloHSCT (NR vs. 14 months; HR 0.61).

A recent retrospective study of 30 younger patients <60 years old with secondary AML, previous cytotoxic therapy, or AML-MRC induced with CPX-351 showed an ORR of 46.7%, CR rate of 17.2%, and CRi rate of 10.3% with an OS of 7 months (0.5–12.4 months) [38]. There is currently a phase 2 single arm study investigating CPX-351 in secondary AML patients <60 years old (NCT04269213), as described below, but randomized studies investigating this approach are needed.

The toxicity profile of CPX-351 is comparable to that of 7 + 3 overall, with febrile neutropenia being the most common adverse event [21]. However, patients treated with CPX-351, as compared to standard 7 + 3, experienced a longer median time to neutrophil and platelet recovery, but no increase in early mortality (5.9% vs. 10.6%) [21].

### 6.2. Hypomethylating Agents (HMAs)

#### 6.2.1. Azacitidine

For many older unfit patients with AML-MRC, the HMAs remain a cornerstone therapy in the management of their disease. In the pivotal phase 3, multicenter, randomized trial AZA-AML-001, azacitidine was compared to several conventional care regimens (CCR), which included 7 + 3 induction chemotherapy, low-dose cytarabine (LDAC), or only supportive care in 488 older patients with newly diagnosed AML [39]. Azacitidine showed increased median OS (10.4 months vs. 6.5 months) with 1-year survival rates of 46.5% compared to 34.2% in all-comers. Furthermore, in the 158 patients with AML-MRC, median OS was 12.7 months for patients treated with azacitidine and 6.3 months for patients treated with CCR (*p* = 0.036).

A central review of bone marrow samples from the AZA-AML-001 study revealed that many more patients carried the diagnosis of AML-MRC than in the original analysis; therefore, azacitidine was further explored in this expanded population of 262 patients [24]. Again, median OS was longer in patients receiving azacitidine compared to those who received CCR (8.9 months vs. 4.9 months, HR 0.74), and 1-year survival was 44.3% vs. 27.2%, respectively. The CR/CRi rate was also improved in the azacitidine group at 24.8% compared to 17.3% in the CCR group.

This study also attempted to determine whether the subtype of AML-MRC or the cytogenetic risk group affected outcomes [24]. Patients with only morphologic multilineage dysplasia had a better OS than all other AML-MRC patients (16.3 months with azacitidine treatment vs. 7.1 months with CCR; HR 0.70, 95% CI: 0.41–1.2), while patients with only MDS-related cytogenetic abnormalities had much shorter median OS (5.3 months with azacitidine treatment vs. 2.9 months with CCR; HR 0.77, 95% CI: 0.47–1.3). A similar result was seen with cytogenetic risk groupings as patients with intermediate-risk cytogenetics had improved median OS following treatment with azacitidine compared to patients with poor-risk cytogenetics (16.4 months vs. 5 months).

#### 6.2.2. Decitabine

Decitabine was initially studied in 485 older patients with newly diagnosed AML in a randomized, multicenter, phase 3 trial comparing decitabine (5 consecutive days every 4 weeks) to treatment choice (TC; LDAC or only supportive care) [40]. In contrast to the encouraging results observed with azacitidine, decitabine failed to demonstrate a significant improvement in median OS compared to TC (7.7 months vs. 5 months; *p* = 0.11). Unfortunately, dedicated analyses pertaining to AML-MRC patients specifically was not performed for this trial, but it did analyze patients with secondary AML separately. In these 171 patients with secondary AML, decitabine failed to show a significant improvement in median OS (*p* = 0.6357), and on multivariate analysis there was no significant difference in OS between de novo and secondary AML (*p* = 0.35) [26,40]. Interestingly, in a planned subgroup analysis of secondary AML patients, treatment with decitabine did show significantly improved response rates (CR/CRi) (*p* = 0.016) [26].

A separate phase 2 trial investigated 10 day cycles of decitabine administered every 4 weeks in patients aged ≥60 [41]. Again analysis of this trial did not separate out AML-MRC patients specifically, but it did include 19 patients (36% of total patients) with secondary or therapy-related AML, of which nine (47%) obtained CR and five (26.3%) obtained CRi for an overall response rate of 74% (95% CI: 49–91%). Furthermore, of 11 patients with monosomy 7 or del(7q), 9 (81.8%) patients achieved a CR.

Although HMAs are commonly employed in the treatment of AML-MRC patients, a number of practical considerations remain. At present, in the absence of head-to-head comparisons, it is not known whether choice of HMA selection matters. However, thus far, combined analysis of the five published phase 3 randomized control trials of HMAs supports an overall survival advantage with azacitidine compared with decitabine (HR for azacitidine 0.67, 95% CI: 0.56–0.79, *p*  <  0.00001; HR for decitabine 0.86, 95% CI: 0.73–1.02, *p* = 0.08) [42]. Additionally, with the recent approval of CPX-351, the decision to use HMAs or CPX-351, particularly in younger (age <60 years) AML-MRC patients, is somewhat controversial and direct comparison studies are needed.

#### 6.2.3. Venetoclax

Venetoclax has recently been approved for newly-diagnosed AML patients ≥75 years, or those unable to tolerate intensive chemotherapy, in combination with either an HMA or low dose cytarabine (LDAC) [43]. The approval of venetoclax-based regimens has revolutionized the management of older AML patients given the high response rates and ease of administration. While the studies leading to this approval did not specifically investigate AML-MRC patients, venetoclax-based regimens offer an attractive treatment option for these patients.

Venetoclax was investigated in combination with HMA therapy in a phase1b open-label, international dose-escalation/expansion study of 145 patients ≥65 years [25]. Patients with secondary AML were allowed, but this study did not permit patients previously treated for myelodysplastic syndrome (MDS). Among all patients, median OS was 17.5 months (95% CI: 12.3-not reached (NR)), and the CR/CRi rates were 37% and 30%, respectively. Encouragingly, patients with secondary AML had the same CR + CRi rate as de novo AML patients (67%), and actually had a longer median duration of CR + CRi (NR; 12.5 months to NR) and median OS (NR; 14.6 months to NR) when compared to de novo AML patients (median duration CR + CRi: 9.4 months; median OS: 12.5 months). This combination still warrants investigation in patients previously treated with HMAs, as they were excluded in this trial, as well as in the other subsets of AML-MRC besides secondary AML.

When studied in combination with LDAC in 82 AML patients ≥60 years, including those previously treated with HMAs for MDS, the CR/CRi rate was 26% and 28%, respectively, with a median OS of 10.1 months (95% CI: 5.7–14.2) [42]. Unfortunately, the 40 patients with secondary AML experienced a lower CR/CRi rate compared to patients with de novo AML (35% vs. 71%, respectively) and only 5% of secondary AML patients achieved a CR. Similarly, the 24 patients previously treated with HMAs fared worse with CR/CRi rate of 33% and median OS of 4.1 months in contrast to a CR/CRi rate of 62% and median OS of 13.5 months in patients without prior HMA therapy. While combination venetoclax with LDAC shows promising results in patients with de novo AML, the benefit of this combination is not especially well supported in AML-MRC patients.

In a separate study, venetoclax monotherapy was also investigated in seven patients with secondary AML (five of which had prior MDS) who relapsed after HMA therapy [44]. Of the patients with prior MDS, one patient achieved CR with progression-free survival (PFS) of 505 days and a second patient experienced peripheral blood blast clearance (did not have bone marrow biopsy) with PFS of 70 days.

The most common and most important side effects venetoclax are myelosuppression, gastrointestinal toxicities, and tumor lysis syndrome. Additionally, venetoclax is a substrate of CYP3A4, and thus dose adjustments must be made when given along with strong or moderate CYP3A inhibitors or P-glycoprotein inhibitors, specifically when antifungal prophylaxis is indicated.

### 6.3. Allogenic Hematopoietic Stem Cell Transplant (alloHSCT)

AlloHSCT is advised for all patients with AML-MRC and remains the only known potentially curative therapy in this patient population [45]. Indeed, alloHSCT may be the “great equalizer” for patients with AML-MRC as alloHSCT has been shown to potentially offset the poor prognostic factors associated with the underlying aggressive disease biology. Two single-institution studies from Japan have demonstrated similar OS, non-relapse mortality (NRM), and cumulative incidence of relapse (CIR) between patients with and without MRC. In the first study by Ikegawa et al., out of 139 patients, 60 patients with MRC experienced similar 2-year OS, CIR, and NRM rates of 48%, 37%, and 19%, respectively, while patients without MRC experienced rates of 59%, 35%, and 13%, respectively [45]. AML-MRC was not an independent prognostic factor for poor outcomes after alloHSCT in multivariate analysis (*p* = 0.7). A second study by Lee et al. of 138 patients showed similar results with no significant difference in 3-year OS (41% vs. 57%), CIR (35% vs. 21%), and NRM (24% vs. 22%) between patients with and without AML-MRC following alloHSCT [46]. Again, multivariate analysis did not find AML-MRC to be an independent prognostic factor (*p* = 0.40) after alloHSCT. Interestingly, MDS-related cytogenetics was shown to be an independent prognostic factor following alloHSCT (HR 4.52, *p* < 0.01), but multilineage dysplasia or history of MDS was not significant. Therefore, patients with AML-MRC who are able to undergo alloHSCT should be encouraged to receive this therapy.

## 7. Future Directions

At present, multiple clinical trials for AML-MRC patients are in progress in an effort to improve responses and outcomes (Table 2). As CPX-351 was originally studied in patients aged 60–75 years, a similar phase 2 study is underway in patients younger than 60 years old with AML-MRC, secondary AML, or t-AML in order to assess the efficacy in this age group (NCT04269213). In a separate phase 2 study, CPX-351 is also being studied in combination with the oral hedgehog inhibitor, glasdegib, in newly diagnosed AML patients who are either ≥75 years or unfit for intensive chemotherapy (NCT04231851). Azacitidine in combination with omacetaxine mepesuccinate, which is currently approved in chronic myeloid leukemia, is being compared to azacitidine alone in an ongoing phase 3 trial in AML-MRC, as well as intermediate and high risk MDS patients (NCT03978364) [47]. Pevonedistat, a small molecule inhibitor of Nedd8-activating enzyme (neural precursor cell expressed developmentally down-regulated protein 8), which may inhibit cell proliferation and survival, in combination with 7 + 3 is being investigated in a phase 1b/2 trial in patients with AML-MRC and t-AML (NCT03330821) [48]. A final study, taking into account that many patients with AML-MRC are elderly with significant comorbidity burden, is implementing a geriatric assessment, along with genetic profiling, to determine treatment for newly-diagnosed AML patients in general, including AML-MRC patients (NCT03226418). Treatment options in this trial include intensive chemotherapy (standard 7 + 3 or liposomal daunorubicin–cytarabine) or low intensity treatment (HMA + venetoclax, HMA alone, low dose cytarabine + glasdegib, or other standard low intensity therapy) and are assigned on the basis of clinical and genetic risk stratification per the trial.

## 8. Conclusions

AML-MRC remains a common and highly aggressive biologic subtype of AML and is associated with poor long-term outcomes. Recent studies have focused on the genetic mechanisms that underlie disease progression in patients with antecedent myelodysplasia as well as towards identification of more tailored therapies for AML-MRC patients. Currently, only CPX-351 is approved for this indication, however, newer treatments for AML have shown promise for AML-MRC patients as well. Future studies directed towards halting leukemic progression in MDS patients are urgently needed, as well as more effective and well-tolerated treatments for those who present with AML-MRC at the time of initial diagnosis.

## Figures and Tables

**Table 1:** Myelodysplasia-related cytogenetic abnormalities (adapted from Arber, D.A., et al. *Blood* 2016.).

Complex Karyotype-3 or More Abnormalities Balanced Translocations
t(5; 10)(q32; q21.2)
t(3; 5)(q25.3; q35.1)
t(5; 17)(q32; p13.2)
t(5; 7)(q32; q11.2)
t(5; 12)(q32; p13.2)
t(2; 11)(p21; q23.3)
t(1; 3)(p36.3; q21.2)
t(3; 21)(q26.2; q22.1)
t(11; 16)(q23.3; p13.3)
**Unbalanced Translocations**
del(12p)/t(12p)
idic(X)(q13)
del(11q)
−13/del(13q)
i(17q)/t(17p)
del(5q)/t(5q)
−7/del(7q)

Del(9q) was removed as a myelodysplasia-related cytogenetic abnormality in the 2016 revision of the WHO classification of myeloid neoplasms and acute myeloid leukemia (AML).

**Table 2:** Current approved therapies and ongoing clinical trials for acute myeloid leukemia (AML) with myelodysplasia-related changes (AML-MRC).

Current Treatment Options for Patients with AML-MRC		
**Treatment**	**Year FDA Approved for AML**	**Outcomes in AML-MRC Patients**
Approved with a specific indication for AML-MRC patients		
Liposomal daunorubicin-cytarabine	2017	CR/CRi rate 48% mOS* 19.15 months
Approved for AML but not with a specific indication for AML-MRC patients		
Standard 7 + 3	First reported in 1973 [49]	CR/CRi rate 33% mOS 11.58 months
Hypomethylating agents (HMAs)		
Azacitidine	2004 (MDS; no specific FDA approval for AML) [50]	CR/CRi rate 24.8% mOS 8.9 months
Decitabine	2006 (MDS; no specific FDA approval for AML) [51]	CR/CRi rate 74% No specific OS analysis
Venetoclax combinations		
Venetoclax with HMA	2018	CR/CRi rate 67% (note: only secondary AML patients analyzed)
Venetoclax with low-dose cytarabine	2018	No specific analysis in AML-MRC group
Allogeneic stem cell transplant	First reported in 1957 [52]	Similar to patients without AML-MRC
**Ongoing Clinical Trials for Patients with AML-MRC ^**		
**Study Title**	**Trial Details**	**NCT Number**
CPX-351 for the Treatment of Secondary Acute Myeloid Leukemia in Patients Younger Than 60 Years Old	Phase 2 Newly diagnosed Includes AML-MRC, secondary AML, t-AML patients	NCT04269213
CPX-351 and Glasdegib for Newly Diagnosed Acute Myelogenous Leukemia With MDS Related Changes or Therapy-related Acute Myeloid Leukemia	Phase 2 Newly diagnosed Includes AML-MRC and t-AML patients	NCT04231851
A Study of Azacitidine for Patients With Int/High -Risk MDS and AML-MRC	Phase 3 Includes intermediate- and high-risk MDS patients and AML-MRC patients with less than 30% blasts	NCT03978364
Pevonedistat, Cytarabine, and Idarubicin in Treating Patients With Acute Myeloid Leukemia	Phase 1b/2 Newly diagnosed Includes AML-MRC and t-AML patients	NCT03330821
Integrating Geriatric Assessment and Genetic Profiling to Personalize Therapy Selection in Older Adults With Acute Myeloid Leukemia	Phase 2 Newly diagnosed Age ≥60 Includes de novo AML, secondary AML, t-AML, other AML equivalent such as myeloid sarcoma, MDS in transformation to AML, or high-grade treatment-related myeloid neoplasm	NCT03226418

* mOS—median OS; **^** all information obtained from clinicaltrials.gov.
